# Acceptability of Physician Directed Academic Detailing to Increase Colorectal Cancer Screening: an Application of the RESPECT Approach

**DOI:** 10.15171/hpp.2015.020

**Published:** 2015-10-25

**Authors:** Gwen Lawson, Corey H. Basch, Patricia Zybert, Randi L. Wolf, Charles E. Basch

**Affiliations:** ^1^Department of Health and Behavior Studies, Teachers College, Columbia University, New York, NY 10027, USA; ^2^Department of Public Health, William Paterson University, Wayne, NJ 07470, USA

**Keywords:** Colorectal cancer, Screening, Colonoscopy, Academic detailing

## Abstract

**Background:** In developing effective interventions to increase colorectal cancer (CRC) screening in at risk populations, a necessary first requirement is feasibility. This paper describes how the RESPECT approach to health education guided the conceptualization and implementation of physician-directed academic detailing (AD) to increase practice-wide CRC screening uptake.

**Methods: ** Physician-directed AD was one intervention component in a large educational randomized controlled trial to increase CRC screening uptake. Study participants, primarily urban minority, were aged 50 or older, insured for CRC screening with no out-of-pocket expense and out of compliance with current screening recommendations. The trial was conducted in the New York City metropolitan area. Participants identified their primary care physician; 564 individuals were recruited, representing 459 physician practices. Two-thirds of the physician practices were randomized to receive AD. The RESPECT approach, modified for AD, comprises: 1) Rapport, 2) Educate, but don’t overwhelm, 3) Start with physicians where they are, 4) Philosophical orientation based on a humanistic approach to education, 5) Engagement of the physician and his/her office staff, 6) Care and show empathy, and 7) Trust. Feasibility was assessed as rate of AD delivery.

**Results:** The AD was delivered to 283 (92.5%) of the 306 practices assigned to receive it; 222/283 (78.4%) delivered to the doctor.

**Conclusion: ** The AD was feasible and acceptable to implement across a range of clinical settings. The RESPECT approach offers a framework for tailoring educational efforts, allowing flexibility, as opposed to strict adherence to a highly structured script or a universal approach.

## Introduction

In the United States, colorectal cancer (CRC) results in approximately 50,000 deaths annually.^1^As screening rates have increased CRC mortality has declined,^2^ but yet it remains the third most deadly cancer in Americans.^1^ Timely, routine screening can prevent CRC incidence and mortality,^3-4^however, screening rates among populations with lower levels of income and education remain sub-optimal.^5^ African American men and women are less likely to be screened for CRC and more likely to be diagnosed at a later stage and consequently suffer from higher CRC morbidity and mortality.^1^ Compared with white patients, non-white patients have been found to be less likely to receive counseling about CRC screening,^6^ despite evidence demonstrating that physician recommendations for screening positively impacts screening uptake among African American patients.^7^

Barriers to physician recommendations about CRC screening may include patient comorbidities, lack of a systematic approach to screening and anticipation of non-compliance.^8-9^ This suggests that both patients and physicians can benefit from targeted education and encouragement of CRC screening.^10^ In fact, evidence suggests that support and encouragement from a primary care provider (PCP), perceived as a trusted source of health information, will influence previously unscreened patients to receive CRC screening.^11-14^

The purpose of this paper is to describe how the RESPECT approach^15^ to health education was applied to physician-directed academic detailing (AD) in a large randomized controlled intervention trial (ClinicalTrials.gov Identifier: NCT023-92143), the Healthy Colon Project 2, (HCP2).^16^ The goal of the AD was increase of practice-wide CRC screening uptake.

## Materials and Methods

The primary objective of the HCP2 was to evaluate the effectiveness of three strategies for promoting CRC screening among low-income, hard-to-reach, men and women, all over 50 years of age, with health insurance, and out of compliance with recommended CRC screening guidelines. The considered strategies were PCP-directed academic detailing (AD), patient-directed tailored telephone education (TTE) and patient-directed mailed educational materials (PEM). Study participants (N = 564) were recruited from a roster of age-eligible enrollees in a self-administered and self-insured benefit fund in the New York metropolitan area. First-dollar coverage (with no deductibles or copayments) for all medically essential services, including CRC screening, was provided by the benefit fund. Study participants identified their PCP, and two-thirds of these PCPs were randomly assigned to receive AD.^16^

In a previous study, we developed an approach to working directly with individuals to help them make an informed choice about CDC screening, which we refer to as the RESPECT approach (described below).^17^ In the current study, we describe how the RESPECT approach was used to conceptualize and implement AD. HCP2’s AD comprised a brief explanation of the project followed by a semi-structured dialogue regarding the PCP’s approach to promote CRC screening. Based upon the PCP’s responses, specific suggestions were offered. The PCP was given a binder of resources that included US screening recommendations, CRC screening research papers,^18-20^ ordering information for printed patient educational materials, communication tips, and patient education material. As appropriate, the detailer tried to elicit a verbal commitment to try one or more new strategies to promote CRC screening. This paper outlines how the RESPECT approach was developed for patient-directed TTE and was applied to PCP-directed AD.

### Enrollment and Randomization

Between February 2011 and January 2013, 564 patients naming 459 physicians as their primary care providers were enrolled and randomized into one of three groups. In one group, patients received printed educational materials sent by mail (PEM). In a second group, patients’ PCPs received academic detailing (AD) intended to improve physician referral and follow up practices regarding CRC screening. In a third group, patients’ PCPs received AD, as above, and patients received tailored telephone education (AD&TTE). A total of 306 PCP’s were assigned to receive AD. PCP office settings varied:  17.6% were located in an apartment building or private home, 24.5% in professional buildings, 47.1% in clinics, 5.9% in hospitals and 4.9% missing data.

### Program Description

The RESPECT approach, modified for AD, includes the following elements: 1) Rapport, 2) Educate, but do not overwhelm, 3) Start with physicians where they are, 4) Philosophical orientation based on a humanistic approach to education, 5) Engagement of the physician and his/her office staff, 6) Care and show empathy, 7) Trust.^15^ Applications of each aspect of the RESPECT approach are described below.

### Rapport

A persistent challenge in delivering ADs was obtaining cooperation for an AD visit with the PCP and scheduling the appointment for a convenient time. If the PCP’s staff discouraged or refused a visit, the detailer employed several strategies to encourage the office to participate: the detailer would explain that the project had no commercial interests, and that the visit would be extremely brief (five minutes or less to suit the PCP’s convenience). If the staff was still hesitant, either because the PCP already had many demands on his or her schedule or because the staff was unsure if they were permitted to schedule such a meeting, the detailer would offer to simply stop by at a convenient time and see if the PCP had a moment to spare, without any hard commitment to hold a meeting. Time was always devoted to getting to know the office staff, and how to suit the doctor’s convenience.

It became evident that visits needed to be scheduled to accommodate long wait-times (typically an hour or more) to see the PCP. This was due to the unpredictability of PCP schedules. If the detailer could not wait as long as necessary to see the PCP, the visit was rescheduled and the binder of resources was left for the PCP. PCP’s schedules could change suddenly due to patient emergencies, walk-in patients, or, in some cases, administrative errors. It was common for the visit to be rescheduled upon arrival, thus taking two attempts to complete.

Meeting busy PCPs required a great degree of accommodation and flexibility so as not to impinge upon their time with patients, a pervasive concern for many physicians. An effort was always made to hear about the physician's concerns (about any part of the project) early on in the conversation. While spending sufficient time with patients was a common worry, there were others as well: worry about special interests, about being unfairly evaluated during the discussion, about the efficacy of the project, about whether or not insurers could ever really be their allies, etc. Hearing PCP concerns at the beginning of the discussion, addressing them straight away and demonstrating that the purpose of the visit was not to lecture or judge them, generally improved PCP’s willingness to engage in a discussion. It should be noted that other than the binder and non-proprietary patient educational materials, no gifts were brought to physicians during these meetings.

### Educate, but do not overwhelm

In order to provide tailored education, the detailer sought to learn in every AD meeting how the PCP addressed patient barriers, whether the PCP used home stool tests and when, and whether the PCP scheduled gastroenterology appointments for their patients. The dialogue was always adapted to suit the PCP’s concerns and needs, and suggestions were made to address the obstacles PCPs faced in implementing CRC screening. Feasibility, based upon factors such as practice size, support staff, and available resources, was a key part of the discussion when suggestions were offered. Care was taken not to overwhelm the doctor with untenable or impractical suggestions. Common concerns and obstacles that PCPs mentioned are listed in Table 1, along with typical detailer responses.

**Table 1:** Obstacles to CRC screening and suggestions offered

Obstacle to Screening	Suggestion Offered
**Patient obstacles**	
Afraid of/resistant to colonoscopy preparation and/or procedure	Emphasize preventive power of colonoscopy; offer home FOBT kits as an alternative
Failure to follow instructions for FOBT kits or return the slides	Use FIT tests instead for less patient preparation
Verbal commitment to screening without following through	Schedule GI appointment for patient
Failure to make routine appointments; general noncompliance with recommendations	Reminder phone calls; discuss screening when such patients come in for a sick visit; emphasize that routine colonoscopy is only repeated every 10 years for average-risk patients
**Provider Obstacles**	
Noncompliance with screening guidelines	Review of current guidelines; provision of printed copy of guidelines; encouragement to take advantage of preventive services
Unsure of which screening guidelines to use	Provision of a table that compares screening guidelines issued by different agencies
Questions about newer tests such as CT colonography, sDNA tests, and CRC blood test	Brief overview of strengths and limitations of a given test; provision of research on those tests when possible
Keeping track of which patients had received the recommendation for screening	Many physicians changed their approach with patients who had been instructed several times to get screened, and still hadn't. This may have involved organizational systems such as stickers, better use of electronic medical records, setting a standard for when it's time to change the way GI screening is recommended, etc., when doctors expressed frustration that many patients receive repeated reminders and still don't get screened.

### Start with physicians where they are

The clinics and clinicians approached varied in many ways: size of practice, site of practice, socioeconomic composition of patient panel, connection to or affiliation with hospital or large healthcare network. Tailoring the AD to the physician’s needs was paramount to the success of AD visits. The needs of a PCP in a small, independent practice differed greatly from the needs of a PCP practicing in a hospital-based office or a clinic with an on-site GI department. Similarly, PCPs who reported that their patients were largely highly educated had different concerns from PCPs who reported that their patients had low levels of education.

PCP ideas and suggestions about encouraging CRC screening in their own practices were noted and, at times, passed along to other PCPs with similar situations. One PCP, noticing that some of his patients needed several discussions before becoming comfortable with the idea of colonoscopy, began discussing CRC screening with patients aged 47 and 48, so that, by age 50, they would be well prepared and knowledgeable. This suggestion was passed on to other PCPs who expressed difficulty motivating their patients to get screened. Several doctors reported tracking patient compliance with screening recommendations so that they knew who needed extra education and encouragement, and possibly additional services such as having the staff schedule the GI appointment. This suggestion was often passed along to other PCPs who expressed interest in making GI appointments for patients but felt they lacked the necessary support staff to do so for all.

### Philosophical orientation based on a humanistic approach to education

PCPs approached for this project varied enormously in terms of office context (e.g.; private practice vs. hospital-based clinic vs. medical group), size of practice, and socio-economic background of patients amongst other factors. Because of these differences, a one-size-fits-all approach was abandoned in favor of a semi-structured conversation that could be tailored to the PCP’s needs and concerns. As with our approach with patients.^16,17^ The conversation typically followed a similar pattern of covering material, with additional time devoted to questions, observations, and concerns broached by the PCP or party receiving detailing. At the same time, the goal was to have a conversation that focused on the unique situation and perspective of each individual physician.

### Engagement of the physician and his/her office staff

AD visits were nearly always arranged through the physician’s office staff. If the staff or the practice policies suggested that the detailer should meet with someone other than the PCP, the detailer would express that a meeting with the PCP would be ideal, but that the meeting could be arranged to suit the convenience of the practice. Sometimes, an AD visit with a third party would lead to a visit with the PCP, once the staff at the clinic determined that this project could be of interest to the PCP.

Though many PCPs welcomed the prospect of an AD visit, some received the visit with some cynicism or suspicion. This was especially true of PCPs who had negative feelings about visits from pharmaceutical representatives, and negative feelings about one or more of the groups conducting the project. Many PCPs also seemed unsure of whether or not the AD visit would be helpful to them and a worthy investment of time. As such, the AD visit was adapted to meet the PCP’s needs and suit his or her communication style. Some typical adaptations to the AD visit in response to the PCP’s affect are listed Table 2.

### Care and show empathy

Because establishing rapport with the PCP and his or her staff was essential to the successful implementation of AD, the detailer made every effort to accommodate the PCP’s schedule and other competing priorities, and to provide materials that best suited the PCP’s patients and practice. In several instances, the PCPs’ staff was reluctant to set up the meeting because the PCP did not see pharmaceutical representatives and the model used to arrange AD was similar to the model used by pharmaceutical representatives.

**Table 2:** Typical adaptations to the AD visit in response to the PCP’s affect

**Affect**	**Response**
Busy/Stressed	Emphasize brevity; shorten AD; finish with office manager or other third party if necessary
Confused	Lengthen project explanation; invite questions
Unhurried	Establish rapport with small talk
Talkative	Ask open-ended questions about attitude toward and approach to CRC screening
Quiet/Reticent	Ask specific questions about patient education, CRC screening tests used, barriers to screening observed in patients, and referral process
Negative/Angry	Determine cause: if PCP is worried about disruption in workday, offer to reschedule or speak with third party; if PCP has concerns about the project, invite questions and provide answers

To ensure that the PCP was making a well-informed decision, the detailer would always explain that the meeting was not a sales meeting and that the project was not for profit. Sometimes, PCPs would express that a nurse or a medical assistant would be the ideal AD recipient, since nurses and medical assistants are often responsible for patient education; such requests were always accommodated, though the detailer always made efforts to speak, at least briefly, to the PCP as well.

PCPs and their staff were offered a variety of patient education materials, and encouraged to select those which best met the needs of their patients. Many PCPs displayed quite a few print brochures and handouts from pharmaceutical companies, and felt that additional print materials would get lost in the clutter. In contrast, some PCPs welcomed education material from public health organizations such as the New York City Department of Health and Mental Hygiene and the U.S. Centers for Disease Control and Prevention, and avoided displaying advertisements in their practices. The majority of PCPs accepted one or more brochures that they felt would meet the needs of their patients. PCPs who felt that brochures and handouts would not suit their patients were offered posters and other materials instead.

Some PCPs noted that commercials seem to have a large impact on patients. When presented with educational brochures, a small number of PCPs expressed a preference for audio-visual education material. PCPs who expressed this sentiment and those with televisions in their waiting rooms were often offered a brief educational DVD about CRC from the American Cancer Society. Few PCPs accepted (N = 6), many explained that their televisions only show pre-programmed material from CNN Access Health, the Healthy Advice Network or other similar services. They were not able to play DVDs, or select the programming themselves.

### Trust

In order to earn the trust of PCPs and their office staff, the detailer made time in every appointment to respond to the PCPs questions about the nature of the project, the sources of funding, and the materials provided. Because so many PCPs feel overburdened with visits and requests from pharmaceutical representatives, many expressed suspicion that the project had commercial interests or would culminate in a sales pitch.

Though nearly every PCP was reassured by the detailer’s explanation that the project was not-for-profit and funded by a grant from the American Cancer Society, a few remained suspicious even after receiving the explanation. One way in which the detailer sought to combat this perception was to provide current, credible, and impartial material for both physicians and patients. All patient-directed material was from reputable public health agencies and non-profit organizations such as the American Cancer Society, the U.S. Centers for Disease Control and Prevention, and the New York City Department of Health and Mental Hygiene. The physician-directed binder included material from these agencies as well as peer-reviewed papers and articles published in highly reputable scientific journals such as the New England Journal of Medicine. In addition, the detailer attempted to build trust by following up with PCPs questions in a timely way. Questions were about reimbursement, general questions about insurance coverage, and whether we could obtain educational materials in other languages. Follow-up on these issues was done in a timely and consistent manner.

### Ethical Considerations

This study was approved by the Institutional Review Board at Teachers College, Columbia University.

## Results

Among the 306 PCP practices that were randomized to receive AD, HCP2 was able to deliver the intervention to 283 (92.5%) and most ADs (222/283 = 78.4%) were delivered to the doctor (either alone or with other staff member). The study encompassed a wide range of PCP practice settings, from offices in private homes to hospital-based clinics. The AD intervention was implemented in all of these various practice settings.

## Discussion

We did not identify any other published studies that examined the feasibility and acceptability of academic detailing to promote CRC screening. The main finding from this paper was that the AD intervention could be implemented in the overwhelming majority of primary care settings (92.5%). The main conclusion is that the physician-directed AD portion of the Healthy Colon Project 2 educational intervention, which was predicated upon the RESPECT approach to health education, was feasible to implement and acceptable within a wide range of health care settings. While the screening rates in all intervention groups were disappointingly low, there was support for the value of AD among the more than two-thirds of patients who actually saw their PCP post-randomization.^16^

This study has several limitations. First, the data were only collected in one geographic region, namely the New York City metropolitan area. Second, we cannot determine the extent to which our ability to successfully reach a very large proportion of the intended population was due to the RESPECT model versus the skills and attributes of the individuals implementing the model.

## Conclusion

This study provides evidence that academic detailing, based on the RESPECT approach, is a feasible and acceptable way to reach large proportion of primary care providers. We do not see academic detailing as a panacea for influencing physicians’ practices related to secondary prevention of CRC or other preventable health problems, but given its feasibility and acceptability, it is a promising strategy to help ensure that proven prevention strategies such as CRC screening are put into practice.

## Acknowledgements

This work was primarily supported by the American Cancer Society (grant number RSGT-09-012-01-CPPB). The authors declare that there are no conflicts of interest.

## Conflict of Interests

The authors declare that there are no conflicts of interest.
